# Study of the permeability of tubular mineral membranes: application to wastewater treatment

**DOI:** 10.1016/j.heliyon.2021.e06837

**Published:** 2021-04-19

**Authors:** Mohammed Messaoudi, Mohamed Douma, Najib Tijani, Lahcen Messaoudi

**Affiliations:** Laboratory of Materials, Membranes and Nanotechnology, Department of Chemistry, Faculty of Sciences, Moulay Ismail University, PB 11201, Zitoune, Meknes, Morocco

**Keywords:** Clay, Tubular membranes, Wastewater treatment

## Abstract

This research work opens up the possibility of developing tubular mineral membranes from Moroccan clay powders and their use in water permeability tests and wastewater treatment. The aim is to show the possibility of using clay as a low-cost raw material for the production of ceramic membranes with high mechanical and chemical performances.

In a first step, we developed ceramic membranes by extruding a prepared plastic paste with the addition of an optimized amount of wood powder as organic matter (OM) to improve the porosity characteristics of the final products after firing. Several parameters are controlled such as the chemical and mineralogical composition of the starting clay powder, the granulometry and the final sintering temperature.

The effect of sintering temperature in the range from 800 to 1000 °C, and OM addition (5, 10, 15wt%) on tubular membrane properties such as mechanical and chemical resistance, porosity and permeability were investigated. It was found that the incorporation of OM in the raw clay enhance the pore volume and the permeate flux but it was also accompanied by a decrease in mechanical strength. The membrane sintered at 1000 °C with 15wt% of OM is considered as optimized membrane and it was applied for the second stage of this work. This stage concerns the treatment of wastewater from a thermal complex located 12 km south of the city of Meknes, Morocco, through a treatment by a biological disk microstation. The filtrate obtained then undergoes tangential filtration by the membranes elaborated and optimized following the evolution of the pollution parameters. Based on physicochemical and biological analyses of wastewater after treatment by the coupled system, the membranes obtained have a good permeability and an excellent pollution removal performance.

## 1 Introduction

Water is very unevenly distributed in the world. The problem of water in the world is not only quantitative; it is also qualitative [1]. Indeed, the increase in industrial and human activity results in significant wastewater discharges that pollute and degrade the aquatic ecosystem [2, 3]. For many years, major efforts have been made to develop and use new, more efficient and less costly treatment processes [4, 5, 6]. Among these processes are the membrane technologies.

Over the past four decades, membrane separation technologies have been the focus of significant research and development efforts [7, 8, 9, 10, 11]. These processes are increasingly used in many and varied sectors such as the chemical industry, the food industry, the pharmaceutical field, biotechnology, the production of drinking water, the treatment of industrial effluents.

Membrane processes are interesting because they have a triple advantage over competing conventional technologies (distillation, separation, extraction...) [3]. Indeed, they are particularly energy efficient, they do not require the addition of any chemical compounds that could lead to downstream processing, and they are faster, more efficient and easily adaptable.

In comparison with polymeric membranes, typically, ceramic membranes exhibit distinguished properties, including high chemical resistance (filtration of organic solvents or aggressive fluids such as acids, bases and oxidants), fairly high thermal resistance (operating temperatures of up to several hundred degrees) and sufficiently high mechanical strength (allowing pressures of around 10–100 bar) [12, 13, 14, 15, 16, 17, 18, 19]. They are available in flat, tubular or hexagonal forms with diameters of more or less important.

Membrane filtration is based on the application of a pressure difference which allows the transfer of solvent through a membrane whose pore size ensures the retention of solutes. Most filtration processes currently used in industry use tangential filtration. Although more costly in terms of energy, tangential filtration has the advantage of limiting the formation of deposits on the membrane, thanks to the shear created by the tangential flow of the fluid on the membrane surface.

The application of ceramic membranes in the field of wastewater treatment is often favored by their resistance to fouling and their chemical stability compared to polymeric membranes [19]. Indeed, the use of membranes is accompanied by a major problem, that of fouling. This phenomenon is the result of the formation of a deposit on the membrane surface. The deposit obtained is generally composed of calcium carbonate, calcium sulphate, silica, bacteria or viruses and biological waste. Fouling modifies the filtering properties of a membrane except for compaction and chemical modification. The ultimate stage is pore fouling leading to both permeability and selectivity variations.

The development of effective fouling control strategies is one of the most active areas of research in membrane science and technology. Among the strategies commonly used to fight fouling in membranes is that of periodic backwashing which improves the permeability of the membrane by reducing fouling within the pores. In this case the influence of backwashing is even more evident than when the filtration flow is high [20, 21]. Photocatalytic materials such as TiO_2_ have also been used for their effectiveness against fouling. They offer a photocatalytic capacity for the decomposition of organic species, microorganisms and pollutants, which reduces undesirable adsorption of these species to the membrane surface [22, 23].

Chemical cleaning of the membrane is also used and remains necessary on a regular basis. This can be done by various methods with varying degrees of effectiveness. The cleaning conditions depend both on the membrane and the nature of the fouling. In this work it is the latter technique that is frequently used.

The cost of the membrane depends on the price of raw materials and the cost of sintering temperature [24]. One of the major challenges of membrane processes is to use/develop membranes from materials that confer new properties to the process [25]. Due to its low cost and abundance, natural clay can present an alternative to inorganic materials commonly used to manufacture ceramic membranes such as titanium, zirconia and alumina oxides [15, 26, 27, 28]. In addition, clay also presents advantage of being densified at relatively low sintering temperatures compared to the oxides mentioned [29].

The main objective of this work is to develop tubular membranes based on a Moroccan clay (as a precursor) and wood powder (as a porogen) in order to evaluate their performances in terms of permeability as a function of time and final sintering temperature and then for the treatment of wastewater from a thermal complex. The first phase of the treatment consists of the degradation of organic matter by a biological disc plant. In a second phase, the water filtered by the plant is subjected to tangential filtration by the membranes previously developed and characterized: it is a question of following the evolution of some pollution parameters before and after each filtration by different membranes treated at different temperatures and at different fractions of organic matter.

## 2 Materials and methods

### 2.1 Raw materials

The choice of clay as a raw material is due to its low cost and its abundance in nature [30, 31, 32]. The clay used in this study was collected from a region in the northwest of Morocco.

### 2.2 Elaboration of clay-based tubular membranes by extrusion process

**Figure 1:**
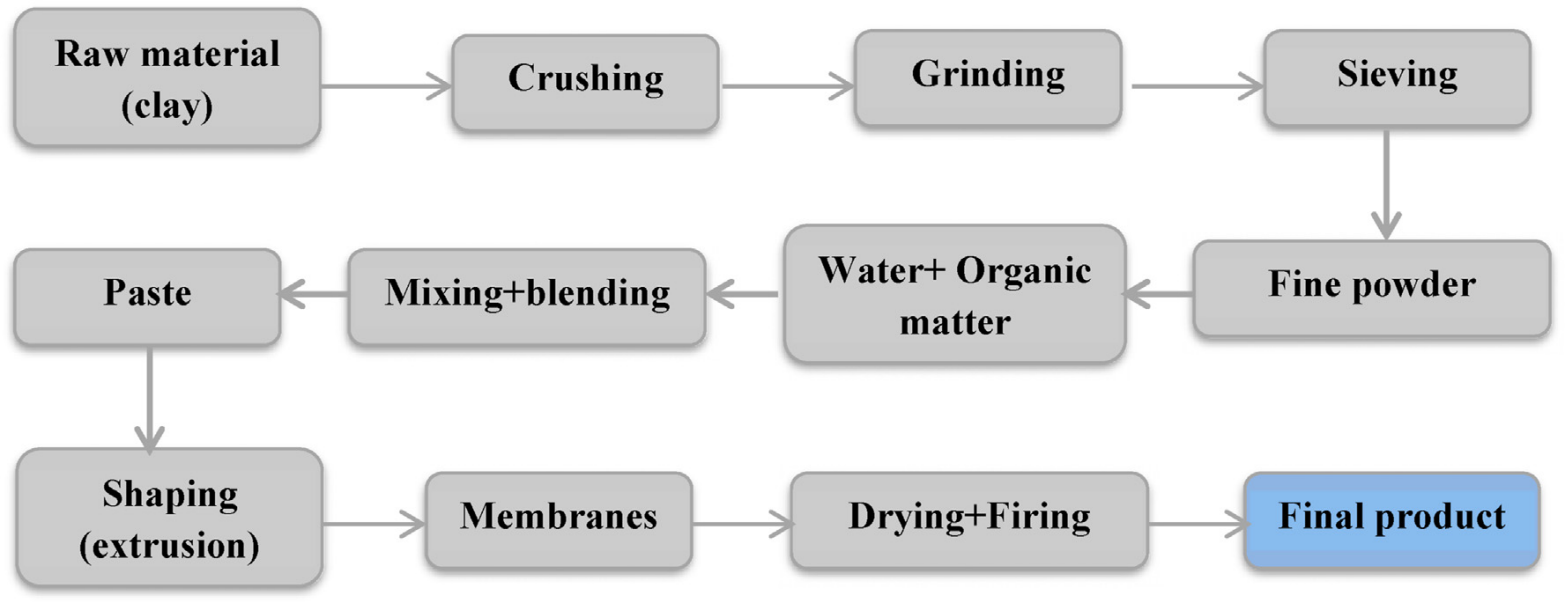
General process for preparing a ceramic piece.

The shaping of tubular membranes by extrusion first requires the preparation of a plastic ceramic paste. Figure 1 shows the different steps followed to obtain these membranes. This shaping is obtained after a large number of trials. We take into account the granulometry of the powder, which is a parameter involved in the sintering process and affects pore size of the membrane [33], and we have chosen to use powders with a size smaller than 250μm.

The final characteristics of ceramic pieces depend on the nature and intrinsic properties of the starting material (clay). Porosity is also related to several factors such as the amount of organic additions, temperature and final sintering time.

The preparation of the paste requires the mixing of clay powder and water with the addition of organic additives to improve the final characteristics of the manufactured piece [15]. The organic additive used in this study is wood powder because of its high availability and lower cost. The added organic wood powder is easily degradable under the action of temperature, hence its contribution to obtaining better porosity after firing [27].

We have thus prepared three paste with different fractions (5, 10 and 15 wt%) of organic matter (wood powder).

❖ 1^st^ paste (3 kg): 5 wt% of organic matter (OM):
(157.89 g of OM + 2842.10 g of clay +700 ml of water)
❖ 2^nd^ paste (3 kg): 10 wt% of the organic matter (OM):
(333.33 g of OM + 2666.66 g of clay +800 ml of water)
❖ 3^rd^ paste (3 kg): 15 wt% of the organic matter (OM):
(529.42 g of OM + 2470.58 g of clay +1000 ml of water)

The prepared paste is then placed in closed plastic bags and stored in a refrigerator for a period of time, called aging time. During this time, the paste becomes homogenized and its qualities are improved by the migration of water. In our case, this time is more than 48 h.

**Figure 2:**
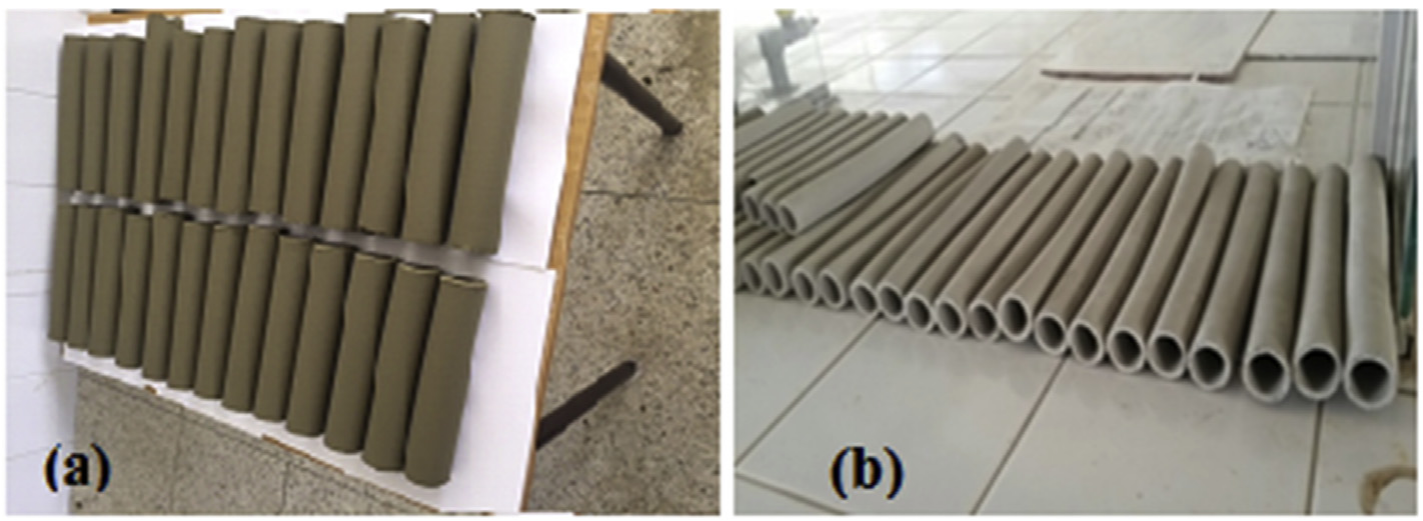
Raw (a) and dry (b) membranes before firing.

After the aging stage, the paste is extruded by a laboratory extruder (Figure 2). The shape of the piece to be obtained depends on the mold used: in our case, we extrude tubular membranes designed for tangential filtration. The extruded pieces are subjected to open air drying for more than 35 h in order to allow time for the water molecules inside to migrate to the outside: this operation is necessary to avoid defects during firing [15].

### 2.3 Heat treatment

It is first necessary to develop an adapted firing program that takes into account the results of the thermogravimetric and differential thermal analyses. This provides information on water discharge, the degradation of organic products and the chemical and structural transformations of mineral materials.

The established program will be made up of stages at the temperatures corresponding to these modifications in order to obtain desired membrane without defaults (cracks and deformation) that are usually encountered in preparation of ceramic membranes. Also the temperature rises must be controlled. The final firing temperature influences sintering, porosity and mechanical strength.

### 2.4 Powder characterization

The clay powder sample must be characterized by different experimental techniques. The chemical composition was determinated by X-ray fluorescence (XRF) using SIEMENS 300, XRD analysis is used in order to identify the crystalline phase of raw clay and it was performed using SHIMADZU 6100 diffractometer employing CuKα radiation (Kα1 = 1,5406 Å), FTIR spectra was carried out using JASCO 4100 spectrometer in the range of 4000–400 cm^−1^ with 4 cm^−1^ of resolution, Thermogravimetric and thermodifferential analysis (TGA and TDA) were carried out using SHIMADZU 60H apparatus under air atmosphere from room temperature to 1000 °C with 10°/min of heating rate. An ASAP2000 Micromeritics apparatus was used to characterize the pore structures and the specific surface area of the raw clay.

## 3 Results and discussion

### 3.1 Characterization of raw materials

#### 3.1.1 XRF analysis

The chemical compositions of the raw clay are shown in Table 1. The average values obtained by X-ray fluorescence show that the clay is rich in oxides of silicon, aluminium and calcium (about 88 wt%), which explains the mechanical performance of this clay. On the other hand, there are very low percentages of alkali and alkaline earth oxides (Na_2_O, K_2_O and MgO) and also the presence of a significant proportion of iron oxide (5,10 wt%) which explains the appearance of the red coloration of the clay after heat treatment [34, 35].

**Table 1:** Main chemical composition (wt%) of clay.

Substance	SiO_2_	Al_2_O_3_	CaO	Fe_2_O_3_	MgO	MnO	Na_2_O	K_2_O	P_2_O_5_	SO_3_	SrO	ZnO	Cr_2_O_3_	TiO_2_
Percent (wt%)	55,61	17,97	14,20	5,10	2,60	0,11	0,67	2,60	0,30	0,10	0,12	0,01	0,02	0,59

#### 3.1.2 XRD analysis

The purpose of the radiocrystallographic study is to determine the mineralogical composition of the studied clay. The measurements are carried out using the powder method. This study allows a qualitative analysis of the minerals present in the dry powder.

**Figure 3:**
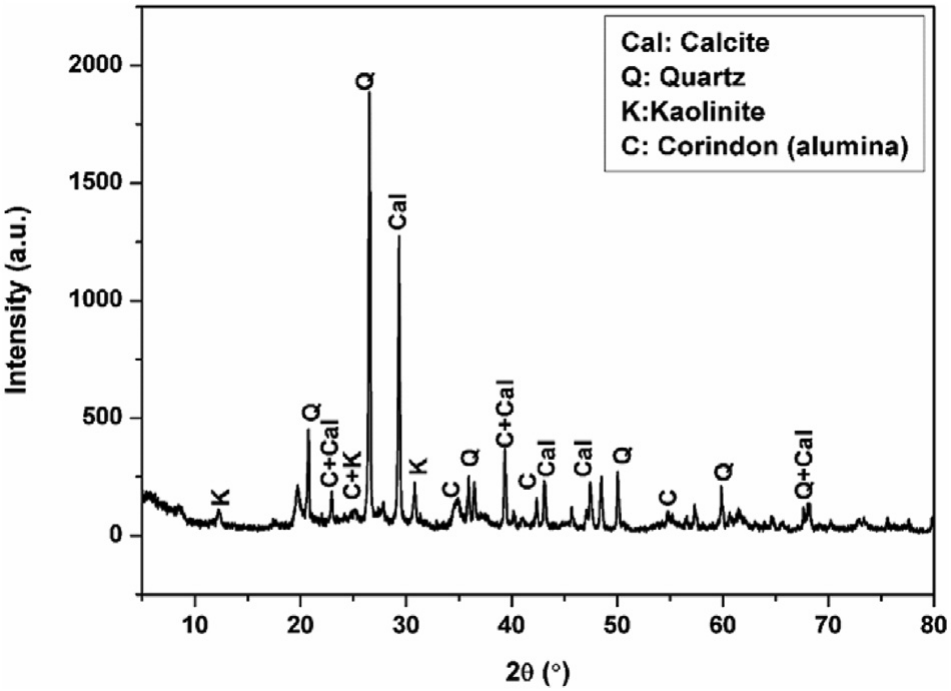
XRD pattern of the clay under study.

Figure 3 illustrates the X-Ray diffraction data of the clay under study. We mainly note the presence of two intense peaks, one corresponding to quartz which is confirmed by the high content of SiO_2_ shown in Table 1 and the other to CaCO_3_. Peaks corresponding to kaolinite Al_2_Si_2_O_5_(OH)_4_ and alumina Al_2_O_3_ are also observed. Peaks diffractions of quartz, calcite, kaolinite and alumina were identified through powder diffractions files *No 98-008-9278*, *No 98-002-0179, No 98-006-8697* and *No 98-016-1790* respectively.

#### 3.1.3 FTIR analysis

**Figure 4:**
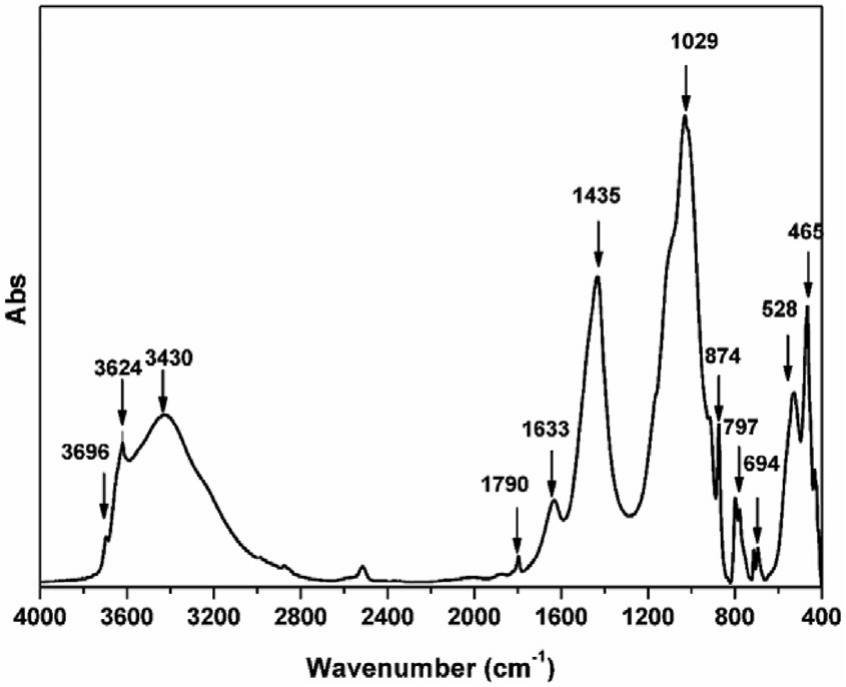
FTIR spectra of the studied clay.

The study of clays by Fourier Transform Infrared Spectroscopy (FTIR) aims to determine the different chemical functions present. It is a complementary technique that generally focuses on the study of samples at the molecular level. The resulting IR spectra are shown in Figure 4.

The different bands recorded in this figure are typical of the characteristic regions of clays [36, 37]. They correspond to the stretching and bending vibrations of the structural hydroxides, organic fraction, carbonates, water of constitution and the (Si, Al, Fe)–O groups.

Three bands are recorded at about 3696, 3624 and 3430 cm^−1^ can be attributed to the O–H bond vibration of hydroxyl groups [38]. The first two bands are attributed to the stretching and bending vibrations of the structural hydroxides of the kaolinite (OH)–Al [39], the third broad band close to 3430 cm^−1^ is assigned to the stretching and bending vibrations of water adsorbed on the surface and between the layers of the clay [40].

We notice some traces of organic matter at 1798 cm^−1^ and at 1633 cm^−1^ which are attributed to the vibration of the C–H and/or C–O bond, which explains the presence of organic matter in the studied clay [41]. We also noted vibrations of the carbonate group (CO_3_^2-^) at 1435 cm^−1^ and 874 cm^−1^, this result is confirmed by X-ray diffraction analysis [42]. The 465 and 1029 cm^−1^ bands attributed to the Si–O–Mg and Si–O–Si stretching vibrations of the clay mineral silicate [25, 42, 43].

The band appeared at 797 cm^−1^ is due to the bending vibration of the Si–O–Al bond while the bands at 528 cm^−1^ and 694 cm^−1^ are attributed to the bending vibrations of Si–O bond in quartz [42, 44].

These results from infrared spectra are in agreement with those found from the XRD diffraction spectra. They confirm the presence of quartz, kaolinite, alumina and calcite in the clay studied.

#### 3.1.4 Gravimetric and differential thermal analysis (TGA-DTA)

**Figure 5:**
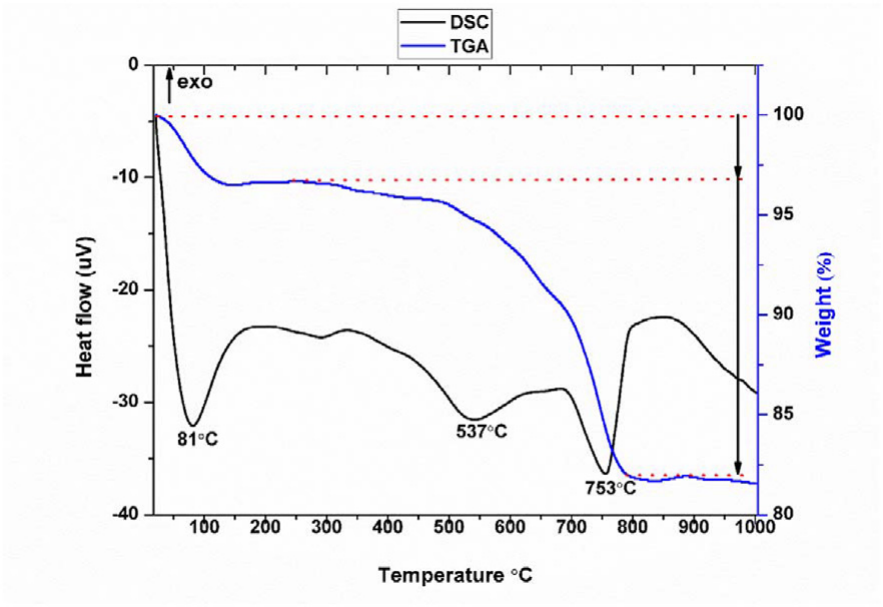
TGA and TDA curves of the studied clay.

Thermal analyses (TGA and DTA) profiles of the studied clay are shown in Figure 5.

The TGA curves (Figure 5) show two main weight losses. The first loss, which begins around 28 °C and ends around 160 °C, corresponds to that of the water absorbed and the second begins around 290 °C and ends around 795 °C, it is mainly due to the dehydroxylation of kaolinite.

❖ A first broad endothermic peak with a maximum at 81 °C associated with a loss of mass, explained by the evaporation of water adsorbed on the surface and inside.
❖ A second endothermic peak appears between 408 °C and 601 °C with a maximum at 537 °C associated with a loss of mass, corresponds to the dehydroxylation of the kaolinite into metakaolinite according to Eq. (1) [45], that means the departure of the water from the structure (the inter-lamellar space).
❖ Another endothermic peak is observed between 678 °C and 815 °C with a maximum at 753 °C. It is associated with a loss of mass due to the decomposition of calcium carbonate.

(1) $${\text{Al}}_{2}{\text{Si}}_{2}{\text{O}}_{5}{\left(\text{OH}\right)}_{4}\to 2{\text{SiO}}_{2},\;{\text{Al}}_{2}{\text{O}}_{3}+2{\text{H}}_{2}\text{O}$$

#### 3.1.5 Textural characterization

**Figure 6:**
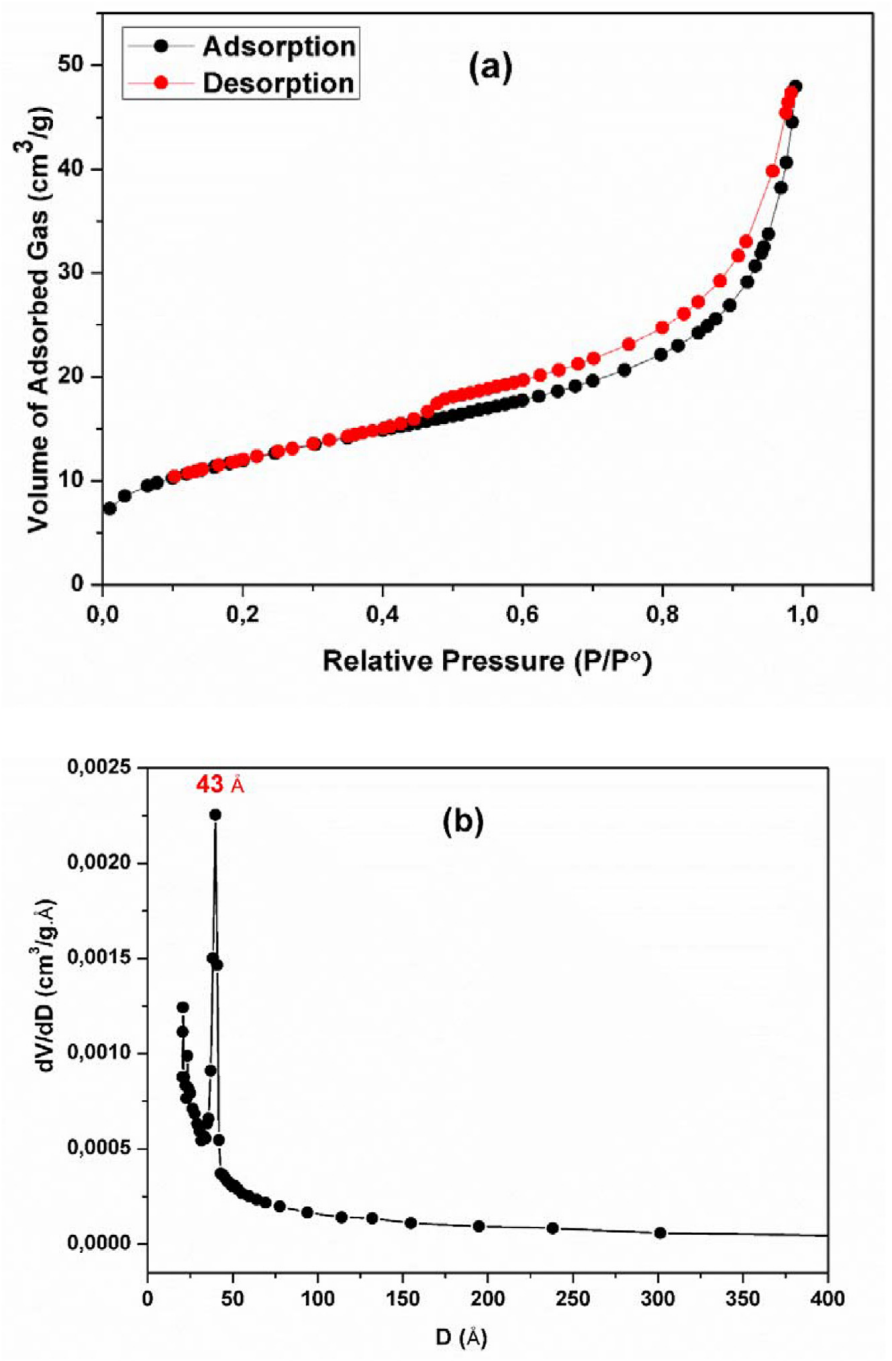
(a) N_2_ adsorption-desorption isotherms and (b) BJH pore size distribution of the raw clay.

The surface area and pore size distribution of the clay studied have been evaluated by N_2_ adsorption-desorption using BET and Barret-Joyner-Halenda (BJH) methods. According to the classification of the International Union of Pure and Applied Chemistry (IUPAC) [46], this sample shows isotherms with profile similar to type IV isotherms (Figure 6a) which is typical of mesoporous structures. The hysteresis loop of the isotherm was H_3_-type, typical of agglomerates of plat-like particles containing slit-shaped pores [47, 48].

The BET specific surface area (S_BET_, m^2^/g) of the raw clay is found to be 42.97 m^2^/g while the total pore volume value was found at about 0.076053 cm^3^/g. The pore size distribution shows a sharp peak concentrated around 43 Å (Figure 6b), and thus is mesoporous.

#### 3.1.6 Heat treatment

The heat treatment thus allows the final consolidation of the shaped piece after removal of water and organic additions. The thermal program must take into account the physicochemical phenomena occurring during differential thermal and gravimetric analysis (DTA, TGA).

The final firing temperatures correspond to good sintering, high porosity and high mechanical resistance. It should be pointed out that sintering does not begin to take place in clays until 680 °C.

The final sintering temperatures (T_F_) chosen are: 800, 900 and 1000 °C.

**Figure 7:**
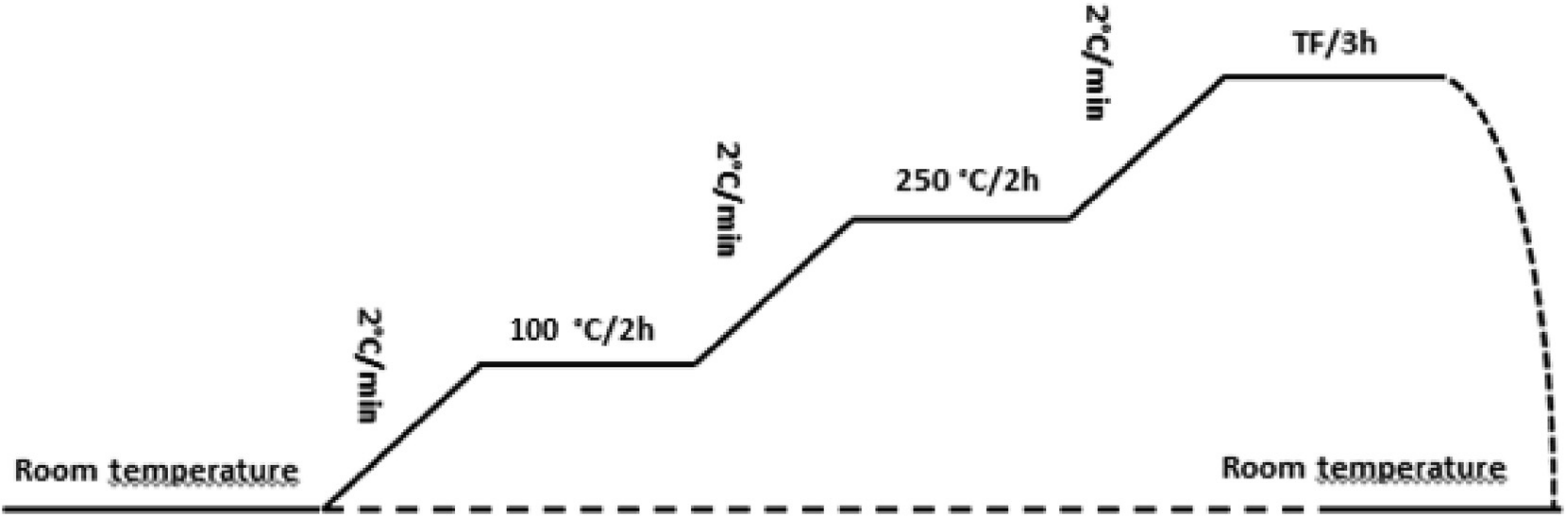
Thermal program used.

The thermal program developed is as follows (Figure 7):

❖ A 2-hour level at 100 °C to ensure the evaporation of the adsorbed water molecules (free molecules).
❖ A 2-hour level at 250 °C for the degradation of organic matter.
❖ A 3-hour level for the final sintering temperatures 800, 900 and 1000 °C, with a rise rate of 2 °C/min.

**Figure 8:**
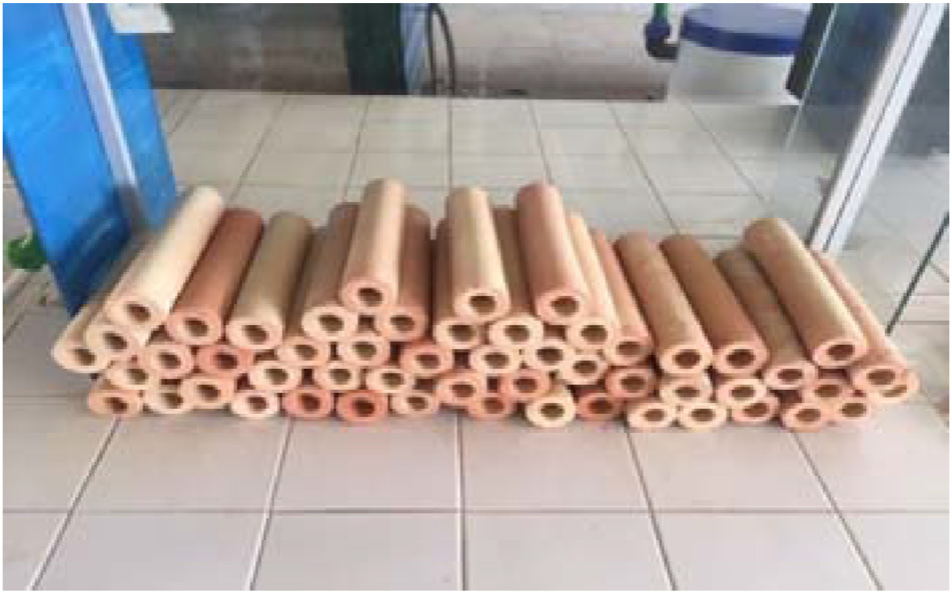
Membranes after firing.

The membranes obtained after firing are shown in Figure 8.

## 4 Membranes characterization

The performance of a membrane is defined by different characteristics such as its selectivity, permeability, manufacturing cost, its resistance to pore fouling as well as its chemical and thermal stability in different working environments [3].

In this paper and after the heat treatment, we proceeded to determine the mechanical and chemical resistance and the porosity.

### 4.1 Mechanical resistance

The flexural strength method was used to measure mechanical resistance of prepared membranes in order to evaluate their ability to resist of hydraulic pressure during filtration. As well known, the mechanical resistance depends mainly on the porosity and the state of sintering material. It was measured by the three point bending test.

The apparatus used at the laboratory scale allows us to apply a force *F* on the sample placed on two supports (two points) until it breaks and we note the maximum breaking force in newton (three-point bending) [25].

The breaking stress is calculated from Eq. (2) [49].

(2) $$\;\sigma =\;\frac{3.F.E}{2.l.e^{2}}\;\left(MPa\right)$$

F: the intensity of the applied force (N)

E: distance between the two specimen supports (mm)

l: width of the sample (mm)

e: thickness of the sample (mm).

Table 2 gives the evolution of the mechanical resistance (σ) of samples treated at 800, 900 and 1000 °C as a function of the fraction of organic matter added.

**Table 2:** Effect of OM on the flexural strength for all membranes sintered at 800 °C, 900 °C and 1000 °C.

wt% of organic matter (OM)	σ in MPa (800 °C)	σ in MPa (900 °C)	σ in MPa (1000 °C)
5	39	40	51
10	38	39	49
15	35	36	49

The mechanical resistance increases with the increase of the final sintering temperature and decreases with the introduction of organic matter. These values show that sintering is best at higher temperatures, so ceramic membrane sintered at 1000 °C for 3 h is the most mechanically resistant.

### 4.2 Chemical resistance

As known, cleaning operation is an important step in the membrane filtration process. There are several cleaning techniques to remove membrane fouling. In a chemical cleaning process, the membranes are soaked in different solutions (acidic or basic mediums) [50], which lead to the rinsing of contaminants. It is for this reason; the study of the chemical resistance is desired to evaluate the chemical corrosion behavior of the tubular membranes.

The chemical resistance tests were carried out in an acidic (HNO_3_, pH = 2) and basic (NaOH, pH = 12.5) mediums by immersing ceramic samples treated at different temperatures and for different fractions of organic matter of known mass under atmospheric conditions for seven days.

**Figure 9:**
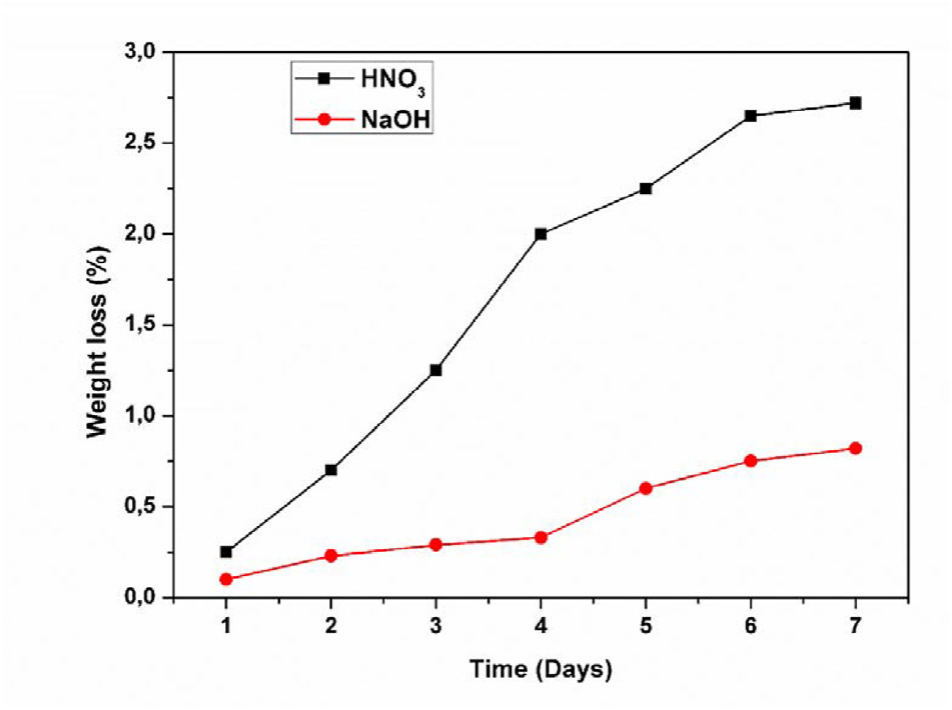
Weight loss versus time curves (at ambient temperature) for the membranes containing 15 wt% of OM and treated at 1000 °C.

After rinsing and drying, the samples are weighed. The evolution of the weight loss of the membranes as a function of time is presented in Figure 9.

These results show that the membranes exhibit an excellent chemical resistance both in acidic and basic mediums, and can be used in filtration operations for industrial liquid effluents. The percentage of loss does not exceed 3% in both mediums.

### 4.3 SEM imaging

**Figure 10:**
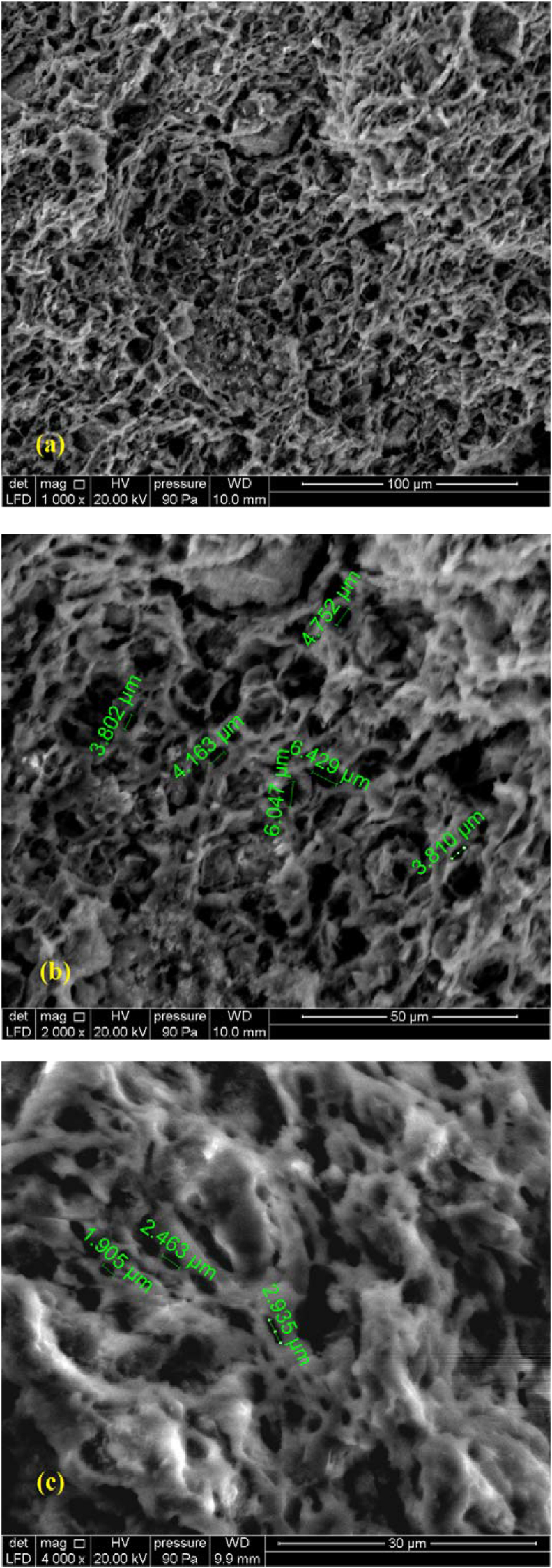
SEM micrographs of the internal surface of the membranes calcined at 1000 °C and containing 15 wt% of OM ((a), (b) and (c) represent different scales and locations).

The Scanning Electron microscopy images were taken by a Jeol JSM 6400 (Tokyo, Japan) (Operating at a voltage of 20 Kv). Figure 10 shows selected SEM micrograph images of the internal surface of the membranes containing 15 wt% of OM and treated at 1000 °C. It can be seen that the distribution of porosity over the entire sample is uniform. The formation of grain boundaries can be easily seen. Moreover, the SEM images shows that there is no defect or crack on the membranes.

### 4.4 Water porosity test

The porosity is an interesting membrane property which not only influences on membrane selectivity, but also on the permeability [51]. The porosity tests consist in immersing the samples in a test tube containing a known volume of hot water (45–50 °C) and the volume of the sample being known, the volume of water occupying the pores of this sample can therefore be deduced: this is the total pore volume Vp.

Table 3 gives the pore volume values for different samples of membranes fired at 1000 °C and at different fractions of organic matter. The pore volume increases with the introduction of the added organic matter. It should be pointed out that after 1000 °C the porosity starts to decrease with temperature due to the matter densification and grain boundaries growth [24, 52, 53].

**Table 3:** Pore volume values for different samples of membranes fired at 1000 °C and at different fractions of organic matter.

T°C	wt% OM	Vp in %
1000	5	15
	10	19
	15	25

The high porosity for the membrane (with 15 wt% in OM) fired at 1000 °C suggests that this one could have important permeability.

### 4.5 Permeation tests

**Figure 11:**
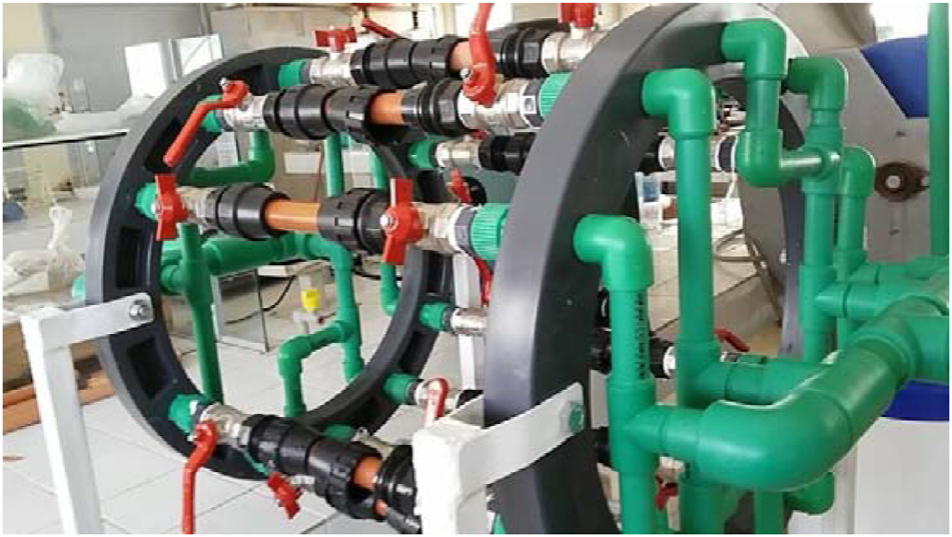
Membrane pilot.

To test the performance of the membranes that we have developed, we opted to carry out a tap water permeation study by mounting the optimized membrane (sintered at 1000 °C) with different fractions of organic matter in a membrane pilot (Figure 11).

The operating mode is such that the water circulates in a closed circuit using an electric circulation pump with a pressure varying between 1 and 6 bars. This is done by recovering the volume of water passing through the membrane (filtrate) in a graduated test tube by determining the time by stopwatch for each working pressure.

The aim is to study the permeate flux as a function of time. The flux values calculated in L⁄h.m^2^, will allow us to draw, for different fractions of organic matter, the curves Flux = f(t) with (t = time in mn) for the optimized membrane (sintered at 1000 °C).

The permeate flux is calculated using the Eq. (3) [49].

(3) $$D=\frac{V}{t*S}\left(l/h.m^{2}\right)$$

With:

D: permeate volume flux (L/h.m^2^);

V: filtrate volume (litre), t: time (hour) and S: filtering surface (m^2^).

The filtering surface of each membrane is calculated using the Eq. (4).

(4) $$\text{S}=2\text{.π.r.l}=\text{π}{\text{.D}}_{\mathrm{int}}.\text{L}\;\left({\text{m}}^{2}\right)$$

D_int_: Internal diameter of the membrane (m)

L: length of the membrane (m).

#### 4.5.1 Permeate flux variation with time

**Figure 12:**
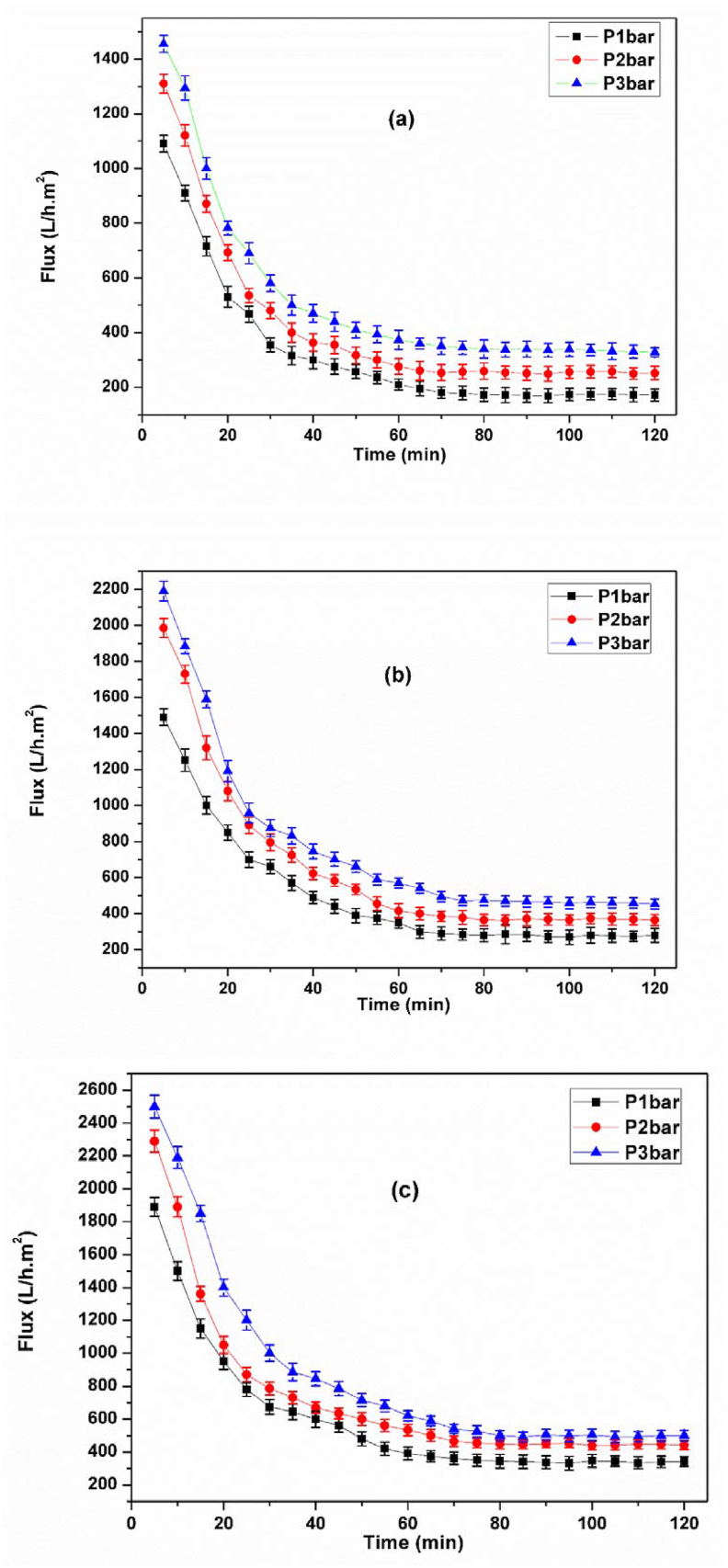
Evolution of the flux as a function of time for the membrane treated at 1000 °C with 5wt% (a), 10wt% (b) and 15wt% (c) of organic matter.

The variation of permeate flux with the time for the membrane treated at 1000 °C for different fractions of organic matter was investigated (Figure 12). The flux values are high for all membranes at the beginning of filtration and then begin to decrease with time until reaching a quasi-stationary regime, this decrease is due to the fouling phenomenon.

## 5 Wastewater treatment

### 5.1 Wastewater extraction

Wastewater is collected from the Ain Salama complex and stored in polyethylene cans. Some parameters are measured in situ, namely: temperature, pH and conductivity. Other parameters are analyzed in the laboratory: Chlorides, sulphates, dissolved oxygen, COD (chemical oxygen demand) and BOD_5_ (biochemical oxygen demand for 5 days).

The experimental protocol consists of the following steps:

❖ Physicochemical and biological analysis of the raw wastewater.
❖ Pre-filtration with sand.
❖ Treatment using a biological disc microstation.
❖ Physicochemical and biological analyses.
❖ Tangential filtration by means of the different membranes developed.
❖ Physicochemical and biological analysis of the filtrate and comparison of the results.

### 5.2 Treatment by biological disk microstation

**Figure 13:**
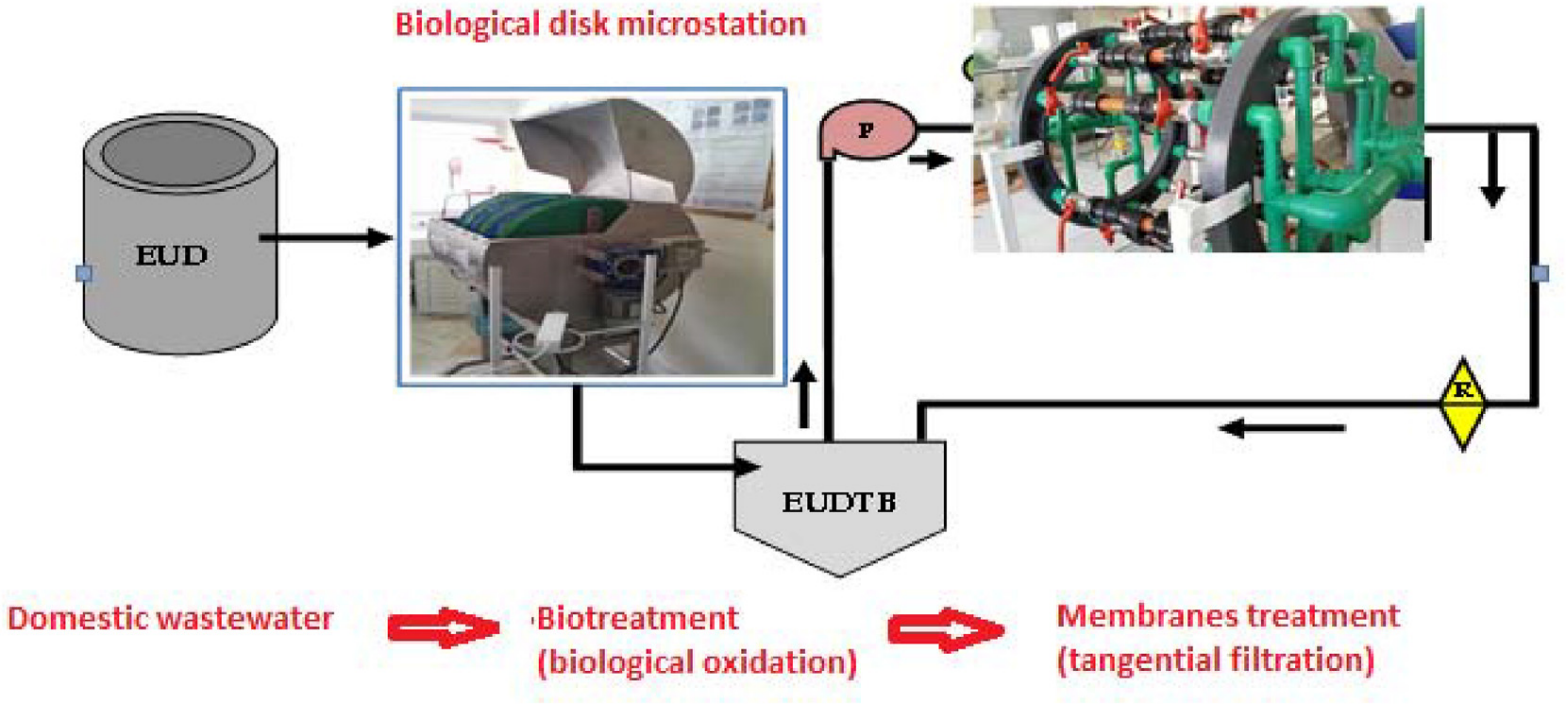
Coupled filtration system developed in the laboratory.

The bio-discs technique is a purification process involving aerobic biological treatment with fixed biomass. The wastewater, after passing over sand, is poured into the tank of the biological reactor and the electric motor is switched on to drive the discs which rotate at a very slow speed, bringing the oxygen from the air which is necessary for the degradation of the organic matter by the bacteria. For this, we developed a coupled system using the biological discs and a membrane pilot (Figure 13).

To ensure the effectiveness of this treatment, we carried out a physicochemical and biological analysis of the filtrate after each filtration.

### 5.3 Pilot membrane filtration

**Figure 14:**
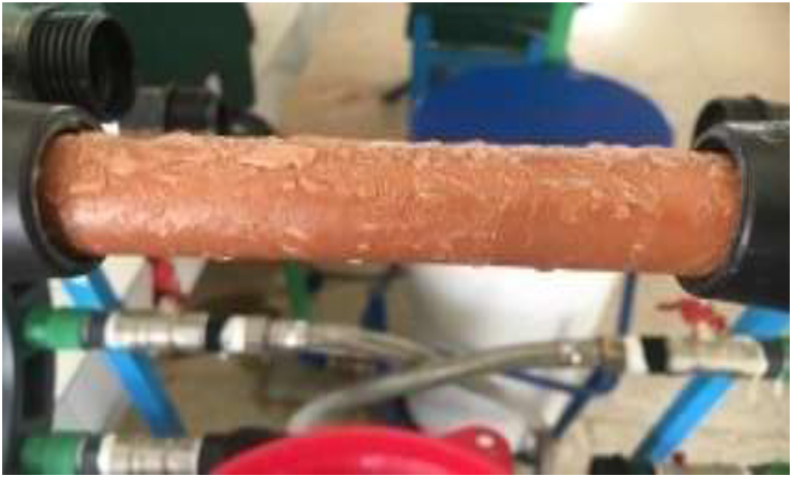
Membrane during filtration.

**Figure 15:**
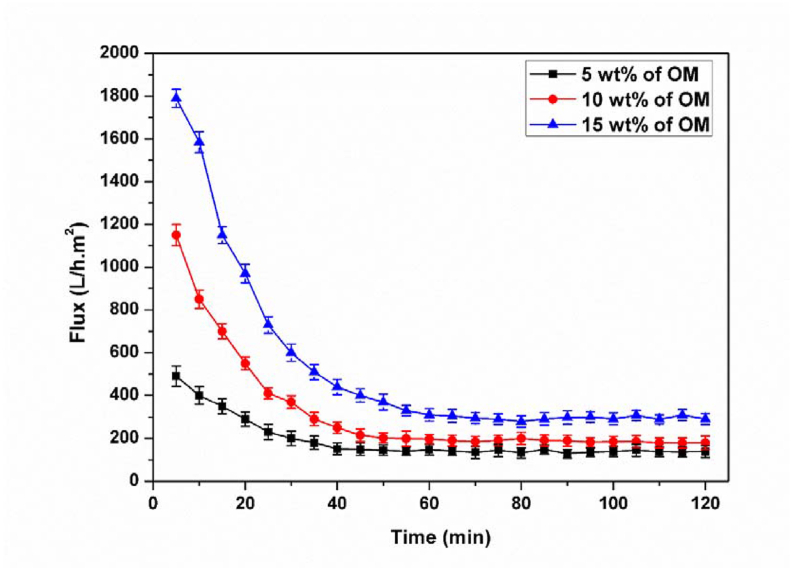
Flux as a function of time (P = 2 bar) for membranes fired at 1000 °C with different fractions of organic matter.

After the biological treatment, the water is discharged into the membrane pilot for tangential filtration (Figure 14). Figure 15 shows the permeate flux evolution as a function of time under pressure of 2 bar during 1h for the optimized membrane fired at 1000 °C and at different fractions of organic matter.

The analysis of the results obtained shows that the flux increases with the increase in the addition of organic matter, with a slight decrease as a function of time. This decrease is due to the fouling of the pores or the formation of a polarization layer by accumulation of particles whose size is of the order of the pores diameter of the membrane [26]. It should be pointed out that after each filtration, the clogged membrane is cleaned by washing it with acid solutions using the vibration method and this test allows us to improve its permeability.

Table 4 reports physicochemical and biological characteristics of the raw wastewater in terms of BOD_5_, COD, pH, Temperature, Chlorides, sulphates and conductivity. These parameters were measured also after filtration by the coupled system in order to investigate the performance of prepared membrane.

**Table 4:** Results of physicochemical and biological analysis of wastewater after treatment by the coupled system.

Parameters	Units	Raw wastewater	After filtration by the biological disc microstation	After membrane filtration
BOD_5_	(mgO_2_/l)	1230	94	41
COD	(mgO_2_/l)	2335	175	85
pH		9,25	8,75	8,45
Conductivity	(us/cm)	2315	1945	945
Temperature	(°C)	21,98	22,5	23,5
Chlorides	(mg/l)	515,4	121,8	35
Sulphates	(mg/l)	21,02	16,65	7,60
Dissolved oxygen	(mg/l)	5,60	2,23	3

According to the results obtained for the temperature before and after treatment, the values are lower than the value considered as limit of rejection and which is 30 °C, and also lower than the value considered to be the limit value for crops irrigation (T = 35 °C) [52].

The decrease in the pH value results from the bacterial activity of the organic matter. The slightly alkaline pH values indicate that the wastewater is necessarily from domestic discharges [53].

Conductivity reflects the overall degree of mineralization, it tells us about the degree of salinity. The decrease in electrical conductivity can be explained by the retention of mineral elements by the membrane. These values are below the standards required for direct discharges of raw wastewater into the receiving environment (=2780 (μs/cm)) [52].

The COD and BOD_5_ values recorded before treatment are high, which can be explained by the presence of organic or mineral fillers dissolved in the water. The decrease in the value of these two parameters shows the effectiveness of the treatment with biological discs.

This reduction is interpreted by the activity of the purifying microorganisms that ensure the degradation and transformation of organic matter, thus allowing the elimination of organic pollution.

The decrease in dissolved oxygen results from the degradation of organic matter by the biological discs. There is also a decrease in sulphate and chloride concentrations between raw and treated wastewater, which can be explained by the efficiency of membrane filtration.

## 6 Conclusion

This study has enabled us to understand a number of phenomena involved in the process of elaboration of tubular ceramic membranes by extrusion, and in the treatment of wastewater by biological discs coupled to the elaborated membranes.

We have shown the possibility of developing cheaper mineral membranes based on Moroccan clay by extruding a prepared plastic paste with the addition of an optimized amount of organic matter to improve the characteristics of the final products. The developed membranes are characterized by different tests, and have been tested for the treatment of wastewater from the Ain Salama complex. Analysis of the results shows that the membranes sintered at 1000 °C with 15 wt% of organic matter have better porosity, good chemical and mechanical resistance, good permeability and excellent pollution removal performance.

The results of the physicochemical analysis of the wastewater allowed us to conclude that the quality of this water is poor and therefore cannot be used without prior treatment. The experimental protocol followed showed the performance of treating wastewater by the biological discs and by the membrane process. Organic matter is practically eliminated and the decrease in pH, conductivity and the concentration of sulphates and chlorides improves the quality of the treated water. We can thus conclude that the filtration membranes developed in the laboratory can be used on a large scale to treat wastewater.

## Declarations

### Author contribution statement

Mohammed MESSAOUDI: Performed the experiments; Analyzed and interpreted the data.

Mohamed DOUMA: Analyzed and interpreted the data; Wrote the paper.

Najib TIJANI: Contributed reagents, materials, analysis tools or data.

Lahcen MESSAOUDI: Conceived and designed the experiments.

### Funding statement

This research did not receive any specific grant from funding agencies in the public, commercial, or not-for-profit sectors.

### Data availability statement

Data included in article/supplementary material/referenced in article.

### Declaration of interests statement

The authors declare no conflict of interest.

### Additional information

No additional information is available for this paper.
